# The Aspirin Regimens in Essential Thrombocythemia (ARES) phase II randomized trial design: Implementation of the serum thromboxane B_2_ assay as an evaluation tool of different aspirin dosing regimens in the clinical setting

**DOI:** 10.1038/s41408-018-0078-3

**Published:** 2018-06-01

**Authors:** Valerio De Stefano, Bianca Rocca, Alberto Tosetto, Denise Soldati, Giovanna Petrucci, Eloise Beggiato, Irene Bertozzi, Silvia Betti, Giuseppe Carli, Monica Carpenedo, Daniele Cattaneo, Viviana Cavalca, Alfredo Dragani, Elena Elli, Guido Finazzi, Alessandra Iurlo, Giuseppe Lanzarone, Laura Lissandrini, Francesca Palandri, Chiara Paoli, Alessandro Rambaldi, Paola Ranalli, Maria Luigia Randi, Alessandra Ricco, Elena Rossi, Marco Ruggeri, Giorgina Specchia, Andrea Timillero, Linda Turnu, Nicola Vianelli, Alessandro M. Vannucchi, Francesco Rodeghiero, Carlo Patrono

**Affiliations:** 1 Institute of Hematology, Catholic University School of Medicine, Fondazione Policlinico Universitario A. Gemelli IRCCS, Rome, Italy; 2 Institute of Pharmacology, Catholic University School of Medicine, Rome, Italy; 3 Hematology Department, Ospedale San Bortolo, Vicenza, Italy; 4 Unit of Hematology, Department of Oncology, University of Torino, Torino, Italy; 5 Department of Medicine-DIMED, University of Padova, Padova, Italy; 6 Hematology Division, Ospedale San Gerardo, ASST Monza, Monza, Italy; 7 Hematology Division, IRCCS Ca’ Granda-Maggiore Policlinico Hospital Foundation and University of Milan, Milan, Italy; 8 Monzino Cardiology Center, IRCCS, Milano, Italy; 9 Hematology Department, S. Spirito Hospital, Pescara, Italy; 10 Hematology Division, Ospedale Papa Giovanni XXIII, Bergamo, Italy; 11 Institute of Hematology “L. and A. Seràgnoli”, S. Orsola-Malpighi Hospital, Bologna, Italy; 12 CRIMM-Center of Research and Innovation of Myeloproliferative Neoplasms, Azienda Ospedaliera Universitaria Careggi, and Department Experimental and Clinical Medicine, University of Firenze, Firenze, Italy; 13 Department of Oncology and Hemato-oncology, University of Milan, Milano, Italy; 14 Department of Emergency and Organ Transplantation (D.E.T.O), Hematology Section, University of Bari, Bari, Italy; 15 Fondazione Progetto Ematologia, Vicenza, Italy

## Abstract

Once-daily (od), low-dose aspirin (75–100 mg) is recommended to reduce the thrombotic risk of patients with essential thrombocytemia (ET). This practice is based on data extrapolated from other high-risk patients and an aspirin trial in polycythemia vera, with the assumption of similar aspirin pharmacodynamics in the two settings. However, the pharmacodynamics of low-dose aspirin is impaired in ET, reflecting accelerated renewal of platelet cyclooxygenase (COX)-1. ARES is a parallel-arm, placebo-controlled, randomized, dose-finding, phase II trial enrolling 300 ET patients to address two main questions. First, whether twice or three times 100 mg aspirin daily dosing is superior to the standard od regimen in inhibiting platelet thromboxane (TX)A_2_ production, without inhibiting vascular prostacyclin biosynthesis. Second, whether long-term persistence of superior biochemical efficacy can be safely maintained with multiple vs. single dosing aspirin regimen. Considering that the primary study end point is serum TXB_2_, a surrogate biomarker of clinical efficacy, a preliminary exercise of reproducibility and validation of this biomarker across all the 11 participating centers was implemented. The results of this preliminary phase demonstrate the importance of controlling reproducibility of biomarkers in multicenter trials and the feasibility of using serum TXB_2_ as a reliable end point for dose-finding studies of novel aspirin regimens.

## Introduction and rationale

Essential thrombocythemia (ET) is a myeloproliferative neoplasm (MPN) characterized by clonal thrombocytosis and enhanced risk of arterial and venous thrombosis^1–3^. The discovery of the *JAK2 V617F* mutation in 2005 and the revised 2008 World Health Organization (WHO) guidelines^4^ indicating a lower platelet count threshold for diagnosing ET, led to an apparent increase in ET incidence^5^. Nowadays, ET incidence approximates 1.0–1.7 per 100 000 individuals per year, with a likely increase in the near future due to the continuous rise of occasional, asymptomatic diagnoses, and an estimated prevalence of approximately 20 per 100 000 individuals^6–8^. ET is usually diagnosed between the fifth and sixth decade, and has a longer life expectancy and a lower leukemic transformation rate as compared to other MPN^1^. However, up to 50% of ET patients experience a thrombotic event, including myocardial infarction, ischemic stroke, transient ischemic attack, or venous thromboembolism^1^, with an estimated incidence of 1.3–6.6% per year in spite of cytoreductive agents and/or antiplatelet drugs^9^. Thrombosis-related mortality in ET approximates 0.5% per year^9^, which ranks higher than the general population^10^. Accordingly, an optimal use of antiplatelet agents seems of outmost clinical relevance.

Several groups have reported increased in vivo platelet activation in ET^11–13^. In particular, we have previously described persistently enhanced urinary excretion of 11-dehydro-thromboxane (TX)B_2_ (TXM) in ET patients^11,12,14^. TXM is the major enzymatic metabolite of TXA_2_ in humans and is largely of platelet origin^15^, therefore its urinary excretion represents a widely used biomarker of platelet activation^9^, which is consistently increased in different clinical settings at high cardiovascular (CV) risk, and predicts CV events in aspirin-treated high-risk patients^16^. Thus, data in ET suggest a pathogenetic link between persistently enhanced platelet activation and thrombotic complications, requiring an effective antiplatelet therapy. Low-dose aspirin (75–100 mg once daily [od]^17^) is currently recommended for both secondary and primary CV prevention in the majority of ET patients^1,9^, with the exception of young patients without traditional CV risk factors, defined at “low risk”, in whom aspirin in primary prophylaxis remains controversial^18^ and possibly dependent on the mutation profile^19^.

The recommendation of using low-dose aspirin in ET patients is mainly based on retrospective, observational analyses^3,9^ and on the extrapolation from an aspirin trial for CV prevention in polycythemia vera^20^. However, controlled trials formally assessing the efficacy and safety of low-dose aspirin in ET are lacking. Thus, the recommendation of the same aspirin dose range (75–100 mg) and dosing regimen (od) for ET patients as for non-ET patients implies assuming similar antiplatelet pharmacodynamics.

The unique pharmacodynamics of low-dose aspirin relies on the irreversible acetylation of platelet cyclooxygenase (COX)-1 and the resulting long-lasting inhibition of TXA_2_ biosynthesis^21^. In spite of aspirin short half-life (20 min in the human circulation), blockade of platelet COX-1 activity lasts for the entire platelet life span due to the limited platelet capacity for new COX-1 synthesis, thus allowing od dosing^21^. Moreover, aspirin acetylates a variable fraction of COX isozymes in the bone marrow megakaryocytes and pro-platelets, as suggested by a 24–48 h delay between aspirin withdrawal and reappearance of COX-1-dependent TXA_2_ biosynthesis in peripheral platelets^22^. Thus, under normal thrombopoiesis, a 24-h dosing interval of a short-lived drug is ensured by a unique combination of irreversible inactivation of a slowly renewable drug target (platelet COX-1) and an effect on platelet progenitors, leading to a new platelet progeny with a largely non-functioning enzyme throughout the 24-h dosing interval^21^. Therefore, at steady state, low-dose aspirin inhibits platelet COX-1 activity by >97% in healthy subjects^22^, as assessed by a surrogate biomarker of efficacy, i.e., the measurement of ex vivo TXB_2_ production during whole-blood clotting^23^.

Low-dose aspirin reduces by ≈25% the rate of major CV events, in a variety of high-risk clinical settings^21,24^. However, at variance with non-ET patients, a standard od regimen of low-dose aspirin administration is inadequate to fully inhibit platelet TXA_2_ production in ≈80% of ET patients^12,14,25^. A faster renewal of the drug target, due to enhanced megakaryopoiesis, is both biologically and pharmacologically plausible in ET^14,26^. Accelerated platelet turnover is associated with a higher-than-normal fraction of newly released platelets with unacetylated COX-1 and/or COX-2^12^, which would account for incomplete inhibition as well as partial recovery of TXA_2_-dependent platelet function during the 24-h dosing interval^9^. Two independent studies have shown that the duration of the antiplatelet effect of low-dose aspirin is shortened in the majority of aspirin-treated ET patients, and incomplete suppression of platelet TXA_2_ production during the 24-h dosing interval can be largely rescued by a twice daily (bid) low-dose aspirin regimen^14,25^. However, approximately one-third of a small group (8 of 22) of ET patients treated with aspirin 100 mg bid still had persistently high serum TXB_2_ values^14^. Interestingly, an increased number of circulating immature platelets represents an independent determinant of poor antiplatelet drug response in non-ET disorders at high CV risk^22^^[,27,28^.

Thus, the abnormal megakaryopoiesis that characterizes ET appears to account for a shorter duration of the antiplatelet effect of low-dose aspirin due to a faster renewal of platelet COX-1, an abnormality that could be rescued by shortening the aspirin dosing interval, but not by increasing the od dose^14,25^. Based on the two small, proof-of-concept studies^14,25^, bid low-dose aspirin is currently considered in the most recent treatment algorithm for low- to high-risk ET patients^1^. However, the clinical efficacy and safety of a bid low-dose aspirin regimen in ET remains to be investigated. Moreover, it should be considered that multiple daily dosing of any drug is usually associated with a lower patient’s compliance^29^. Although a bid low-dose aspirin regimen has been successfully tested in stroke patients^30^, nevertheless this issue should be addressed when proposing multiple daily drug intake for further clinical evaluation.

The potential inhibitory effect of aspirin on vascular prostacyclin (PGI_2_) biosynthesis should also be considered. In fact, the COX-2 isozyme constitutively expressed in vascular endothelial cells largely accounts for PGI_2_ biosynthesis under physiological shear conditions^31^. In humans, PGI_2_ has vasodilator and platelet-inhibiting effects, counteracting pro-thrombotic signals, including platelet TXA_2_^31^. Od low-dose aspirin within the low-dose range has limited inhibitory effects on in vivo PGI_2_ biosynthesis, while it fully inhibits platelet TXA_2_ production, possibly because of differential rates of recovery of endothelial COX-2 vs. platelet COX-1 during the 24-h dosing interval^21,32,33^. It is unknown whether shortening the aspirin dosing interval may affect endothelial PGI_2_ production. A pilot study in 50 ET patients suggests that aspirin 100 mg bid does not significantly affect PGI_2_ biosynthesis^33^. However, the potential impact of a shorter dosing interval of low-dose aspirin administration on in vivo PGI_2_ biosynthesis should be investigated.

To address the open questions outlined above, we designed the Aspirin Regimens in Essential Thrombocythemia (ARES: EudraCT 2016-002885-30) trial as a phase II dose-finding study of aspirin in ET to select the optimal dosing regimen for an international phase III trial in ET. The ARES trial has been approved and funded by the Italian Medicines Agency (AIFA), study code FARM12Y8H.

## Study objectives

The ARES study has two primary objectives:

1. To investigate whether aspirin regimens based on bid or three times daily (tid) administration of 100 mg result in a more complete suppression of platelet-derived TXA_2_ throughout the dosing interval, without significantly affecting in vivo PGI_2_ biosynthesis, as compared to the standard od regimen. Serum TXB_2_ will be measured as an index of platelet COX-1 activity, specifically reflecting the antiplatelet pharmacodynamics of aspirin (biochemical efficacy) (http://www.ema.europa.eu/docs/en_GB/document_library/Scientific_guideline/2009/09/WC500003340.pdf). A major urinary PGI_2_ metabolite, 2,3-dinor-6-keto-PGF_1alfa_ (PGIM) will be measured to assess the impact of different aspirin regimens on vascular COX-2 activity (biochemical safety)^32^. The comparison between aspirin 100 mg bid or tid vs. 100 mg od will test a superiority hypothesis in terms of serum TXB_2_ levels associated with each experimental vs. standard regimens. PGIM comparisons will assess the non-inferiority of any multiple daily dosing regimen vs. the standard od regimen. This objective will be addressed by a randomized, parallel-arm, double-blind, controlled study of 2-week aspirin treatment (part A) aimed at identifying the aspirin regimen to be further evaluated during long-term follow-up in the second part (part B) of the study.
2. To evaluate the long-term persistence of superior biochemical efficacy of an optimized, multiple daily dosing regimen, as compared to the aspirin 100 mg od regimen. Biochemical efficacy will be assessed by repeated measurements of serum TXB_2_ (every 3 months over 20 months). A multiple daily dosing regimen will be tested for superiority vs. od dosing in terms of biochemical efficacy throughout the dosing interval, in an open-label, randomized study comparing aspirin 100 mg od vs. the optimal multiple daily dosing regimen identified in part A, with a follow-up of 20 months. This long-term follow-up will also provide an estimate of compliance with the experimental dosing regimen.

The secondary exploratory objectives will be:

1. To assess the safety of the multiple daily aspirin regimen by recording: major bleeding and clinically relevant non-major bleeding events defined according to the Scientific and Standardization Committee of the International Society on Thrombosis and Haemostasis^34,35^, as well as any upper gastrointestinal non-bleeding adverse events, which may be considered attributable to aspirin (e.g., ulcer or perforation > grade 2).
2. To record any thrombotic complication, as previously defined^3^. Briefly, major arterial thrombosis will include the following: acute coronary syndrome; any ischemic stroke (major and minor); and peripheral arterial thrombosis, including thrombotic digital ischemia and retinal arterial thrombosis. Major venous thrombosis will include thrombosis in the following districts: deep veins of the limb and/or abdomen; cerebral and splanchnic veins; retinal vein, as well as pulmonary embolism. Splanchnic venous thrombosis will include hepatic, portal, mesenteric, and splenic veins. Transient ischemic attack and superficial vein thrombosis of the limbs will be considered as minor thrombosis.
3. To assess the tolerability of the experimental dosing regimen by recording the gastrointestinal symptoms by the severity of dyspepsia assessment questionnaire^36^.
4. To evaluate the potential benefit of multiple doses of aspirin on the MPN-related symptom burden by a questionnaire aimed to capture all microvascular symptoms^37^, including the MPN Symptom Assessment and a pain numeric rating scale for erythromelalgia.
5. To assess the stability over time of in vivo platelet activation, as assessed by urinary TXM excretion, in a subset of patients, in a non-invasive substudy.
6. To assess whether the pre-fibrotic/early primary myelofibrosis (pre-PMF) phenotype now distinguished in the revised 2016 WHO classification^38^ has a higher incidence in the patients who will develop major or clinically relevant non-major bleeding during follow-up.

These secondary assessments will be performed in part B of the study, over 20-month treatment.

## Design of the study

**Fig. 1:**
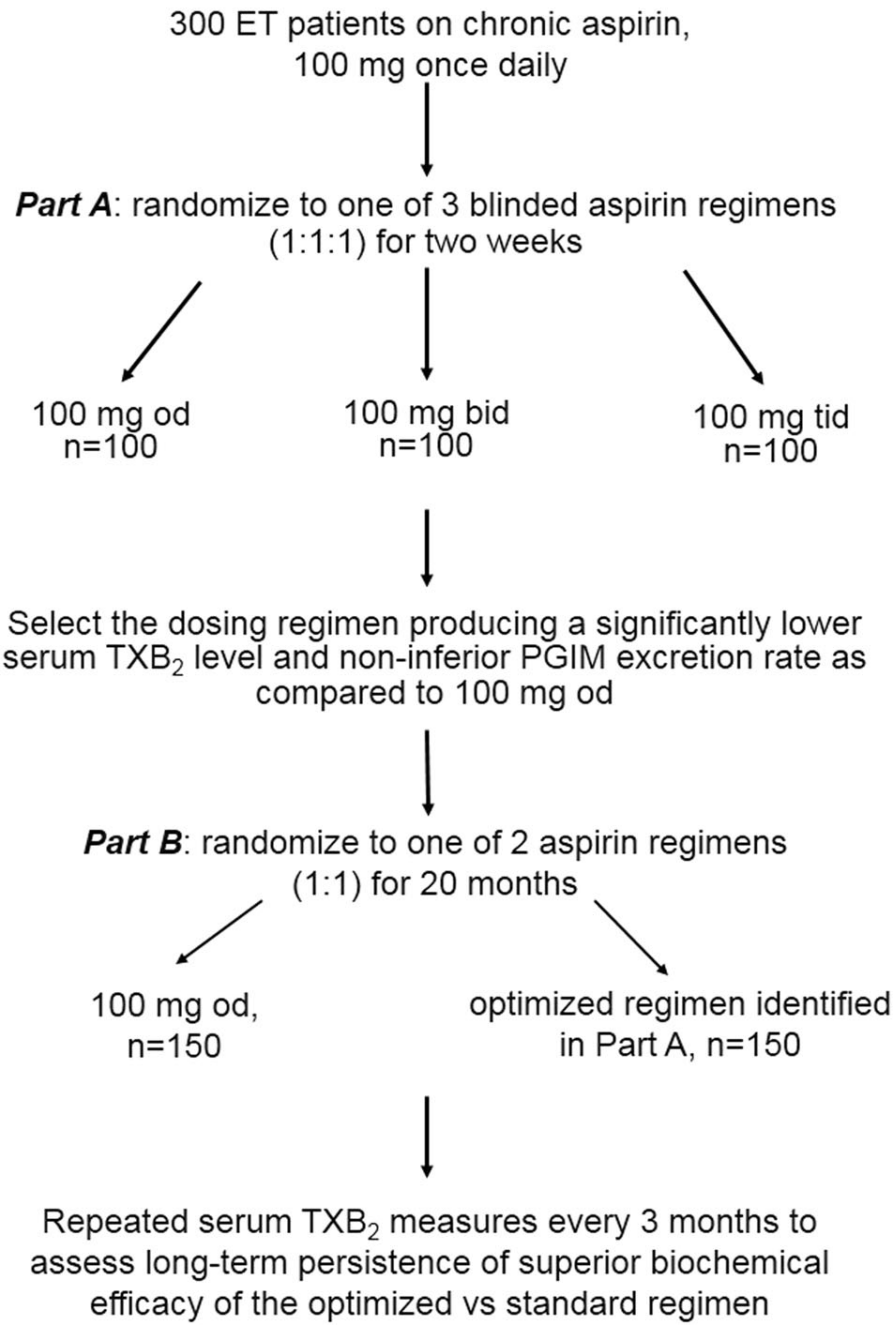
Trial design. The flow chart depicts the design and phases of the ARES study

The ARES study consists of two sequential parts, “A” and “B” (Fig. 1). Three-hundred ET eligible patients, after signing an informed consent, will start a run-in phase, whereby they will be instructed to take their aspirin tablet at breakfast (7–9 a.m.) for 7–10 consecutive days, thus allowing synchronizing aspirin intake. Upon run-in completion, patients will enter study part A.

### Part A

Patients will be randomized (1:1:1) in a double-blind fashion to aspirin 100 mg od (standard of care), 100 mg bid (i.e. breakfast and dinner), or 100 mg tid (i.e., breakfast, lunch, and dinner). Matching placebo will be used so that all patients will take active drug and placebo tablets tid. At randomization and after 2 weeks of study treatment, patients will undergo blood and urine sampling for serum TXB_2_ and urinary PGIM and TXM measurements at 8 a.m., immediately before aspirin dosing; thereafter, they will resume their open-label, standard aspirin regimen for the time interval necessary to assay serum and urine samples and analyze the data. The primary end points of part A will assess the biochemical efficacy, as reflected by the degree of suppression of serum TXB_2_ throughout the dosing interval, and biochemical safety, as assessed by urinary PGIM excretion, of the two experimental dosing regimens as compared to the standard regimen of aspirin administration. The secondary end point will assess their impact on in vivo platelet activation, as reflected by the urinary excretion of TXM.

### Part B

The experimental aspirin regimen associated with a significantly lower serum TXB_2_ level and non-inferior urinary PGIM excretion rate (i.e., ≤30% reduction) as compared to aspirin 100 mg od, will be selected for part B, and patients will be randomized in an open-label fashion to the standard vs. the optimized multiple dosing regimen for 20 months. The primary end point of part B will assess the long-term persistence of the superior biochemical efficacy of the optimized vs. standard aspirin regimen, in at least 6 out of 10 determinations of serum TXB_2_ that will be performed over 20 months. Secondary end points of part B will explore the following: (i) the safety of the experimental aspirin regimen, as reflected by any major bleeding and gastrointestinal symptoms considered attributable to aspirin; (ii) effectiveness in reducing MPN-specific symptom burden and pain attributable to microcirculatory disturbances; and (iii) stability over 20 months of the degree of platelet activation in vivo, as assessed by urinary TXM. The stability of TXM will be assessed in a subgroup of 150 patients.

## Study population and patient eligibility

Three-hundred ET patients will be enrolled by 11 Italian hematological centers. Both patients with newly diagnosed and previously diagnosed disease were eligible. Inclusion and exclusion criteria are listed in Table 1. The following characteristics will be recorded at study entry: age at diagnosis; history of thrombosis or major bleeding; mutational profile (i.e., JAK2, CALR, and MPL mutations); blood count; spleen size; constitutional symptoms; and cytoreductive agents. Of note, the study was designed and approved by the AIFA and Ethic Committees before the publication of the revised 2016 WHO classification for tumors of the hematopoietic and lymphoid tissues^38^, therefore the inclusion criteria reflect the WHO classification at the time of study approvals.

**Table 1:** Inclusion and exclusion criteria

Main inclusion criteria	Main exclusion criteria
*All of the following*: Age between 18 and 75 years A WHO 2008-defined ET diagnosis Ongoing aspirin 100 mg daily since at least 3 months, according to the judgment of the referring hematologist The patient understands and voluntarily signs an informed consent	*Any of the following*: Platelet count > 1 000 000/μl on three occasions over the 2 months before enrollment Diabetes according to American Diabetes Association criteria Creatinine level > 1.5× upper limit of normal Liver disease defined as AST and/or ALT values > 3× upper limit of normal Active gastrointestinal disease Obesity (BMI > 30 kg/m^2^) Smoking habits (>5 cigarettes/day) History of major bleeding History of cancer in the previous 3 years, except for treated early-stage squamous or basal cell skin carcinomas Pregnancy or lactation Use of nonsteroidal anti-inflammatory drugs >3 times/week Use of antiplatelet agents other than aspirin 100 mg Use of oral anticoagulants including anti-vitamin K, anti-Xa, or -IIa agents Use of heparins or fondaparinux Chronic use of steroids (prednisone > 5 mg/day or equivalent)

Cytoreductive drugs, namely hydroxyurea, pipobroman, busulphan, interferon, and anagrelide will be allowed to control platelet count. Patients will be prescribed proton pump inhibitors according to the Italian regulatory indications. In case of the occasional need of analgesic/antipyretic drugs, patients will be instructed to take paracetamol (up to 2000 mg daily) and to avoid traditional nonsteroidal anti-inflammatory drugs (NSAIDs) known to have a pharmacodynamic interaction with low-dose aspirin that may limit the extent of platelet COX-1 acetylation^21^. Patients will be instructed to take paracetamol for a maximum of 3 days/week, if necessary

## Study end points and statistical analysis

The co-primary end points of part A are as follows: (1) platelet TXA_2_ production ex vivo, as reflected by serum TXB_2_, measured in samples collected in the morning, before the next aspirin intake; and (2) vascular PGI_2_ biosynthesis in vivo, as reflected by urinary PGIM excretion in a urine sample collected in the morning before the next aspirin intake. Urinary TXM excretion represents a secondary end point. These biomarkers will be measured at randomization and at 14 ± 2 days thereafter.

The primary end point of part B is represented by serum TXB_2_ measured 10 times in samples collected in the morning, before the next aspirin intake. The secondary end points are related to exploratory assessment of safety and tolerability of the experimental aspirin dosing regimen, and stability over time of in vivo platelet activation, as detailed above.

Based on previous findings^12,14^, we expect the mean ± standard deviation (SD) serum TXB_2_ in ET patients on aspirin 100 mg od and 100 mg bid to be 22 ± 33 and 5.0 ± 6.0 ng/ml, respectively. We plan to test with *α*-error of 0.05 and a *β*-error of 0.2 (power 80%) the following hypotheses:

- 100 mg bid is superior to 100 mg od, with a ≥50% reduction in serum TXB_2_ (required sample size 70 patients/arm)
- 100 mg tid is superior to 100 mg bid, with a ≥50% reduction in serum TXB_2_ (required sample size 70 patients/arm)

Anticipating a 30% dropout over the entire study duration, we plan to enroll 100 patients/arm to ensure adequate statistical power. For urinary PGIM, we expect the mean ± SD PGIM excretion rate in ET patients on aspirin 100 mg od to be 195 ± 119 pg/mg creatinine^33^. Using the above sample size (*n* = 70 patients/arm), the study has 80% power to test the hypothesis that any experimental treatment may reduce urinary PGIM to <140 pg/mg creatinine, i.e., by >30%. This threshold of PGIM inhibition vs. the standard dosing regimen can be considered reasonably safe based on the following considerations: urinary PGIM excretion is minimally affected by low-dose aspirin in healthy subjects^32,33^; in ET subjects, aspirin 100 mg bid did not significantly modify PGIM as compared to 100 mg od^33^; and this threshold corresponds to the intra-subject coefficient of variation on repeated measurements of PGIM excretion over time^39^.

The same 300 ET patients will be randomized in part B of the study that will test the long-term persistence of superior biochemical efficacy of the optimized vs. standard dosing regimen. In all, 112 patients/arm will be needed to assess with an *α*-error of 0.05 and 80% power, a reduction of at least 50% in serum TXB_2_ with the optimized regimen (100 mg bid or tid) vs. the standard aspirin regimen (100 mg od), in at least 6 out of 10 determinations performed over 20 months.

Differences in mean serum TXB_2_ values will be evaluated by one-way analysis of variance, using Scheffe multiple-comparison test to allow comparisons of the three different treatments in part A. Analysis of covariance using multiple regression with dummies for the different treatments will be used if, at single univariate analysis, major differences (*p* < 0.05) in the distribution of gender, age, platelet count, JAK2 mutational status, spleen size, aspartate aminotransferase, alanine aminotransferase, or creatinine, and type of cytoreductive drug (if any) will be present between the three treatment subgroups. Both intention-to-treat and per-protocol analyses will be carried out.

It can be reasonably anticipated that a portion of the ET patients recruited in this trial according to the 2008 WHO diagnostic criteria^4^ might fall into the category of the pre-PMF according to the revised 2016 WHO criteria^38^. In a large international study of 1104 ET patients, diagnosis was revised to pre-PMF in 16%;^40^ these patients have been reported having an increased tendency toward bleeding^41^. Therefore, we will perform a pre-specified secondary analysis in the group of patients who will develop major and/or non-major clinically relevant bleeding in comparison with the patients with an uneventful course. All the bone marrow biopsies of the recruited patients will be revised by an ad hoc committee formed by the pathologist of the Coordinating Center and the pathologist of the Center where the patient had been recruited in order to assess whether patients had a true-ET or a pre-PMF according to the revised WHO classification;^38^ both the pathologists involved in the bone marrow revision will be blinded to the clinical characteristics of the patients. If the pathologists will provide different opinions, we will consult with a qualified third pathologist. The incidence of pre-PMF in patients with bleeding events will be compared to that found in non-bleeders.

## Study organization: feasibility and implementation of the serum TXB_2_ assay

The measurement of TXB_2_ generated ex vivo during whole-blood clotting at 37 °C is a highly specific biomarker to characterize the pharmacodynamics of low-dose aspirin as an inhibitor of platelet COX-1^23,42^. This assay relies on the physiological generation of endogenous thrombin during whole-blood clotting at 37 °C, which triggers the release of arachidonic acid from platelet membrane phospholipids^43^. Arachidonic acid is then metabolized by COX-1 to the unstable intermediates prostaglandin (PG)G_2_ and PGH_2_, which is converted to TXA_2_ by TX-synthase^21^. TXA_2_ is not a circulating substance (max estimated plasma concentration: 1–2 pg/ml)^15^, is rapidly hydrolyzed to TXB_2_ in an aqueous milieu, and its abundant presence in serum (300–400 ng/ml in the absence of aspirin) reflects its platelet COX-1-dependent biosynthesis during whole-blood clotting, as the end product of a chain of enzymatic reactions that are both time- and temperature-dependent^23^. Thus, serum TXB_2_ reflects the maximal biosynthetic capacity of blood platelets to generate TXA_2_ in a COX-1-dependent fashion. This assay was used to characterize the clinical pharmacology of platelet COX-1 inactivation by low-dose aspirin in health and disease^44^.

In order for the serum TXB_2_ assay to reflect the maximal biosynthetic capacity of blood platelets and its blockade by COX-1 inhibitors in a reproducible fashion, initiation of whole-blood clotting at 37 °C must rapidly follow peripheral blood sampling. However, a reproducible implementation of this procedure in multicenter studies might face practical hurdles, such as logistic delays between blood withdrawal from patients and access to a thermostatic bath, as well as the lack of appreciation of the time- and temperature-dependence of TXB_2_ production during blood clotting. Consistent with this expectation, a comparison of serum TXB_2_ values in two large, multicenter cohorts of aspirin-treated patients^45,46^ showed up to 10-fold difference in median TXB_2_ levels (7 and 0.6 ng/ml in the ADRIE^46^ and BOSTON^45^ studies, respectively) that could not be explained by patient characteristics or analytical biases^47^. Two recent in vitro studies showed that even a minor delay in starting 37 °C incubation can time-dependently underestimate serum TXB_2_ levels^17,48^, and thus potentially account for variable aspirin responsiveness across studies and centers. Thus, we assessed the feasibility of obtaining reliable serum TXB_2_ measurements across the ARES study centers. All participating investigators were given a detailed operative manual for the pre-analytical procedures, and all centers were supplied with the same disposable material for collecting and processing blood. Each center recruited five healthy, non-smoker subjects not being treated with any medication, and with normal hematochemistry, who did not take any NSAID or aspirin in the previous 10 days. The reason for including healthy, aspirin-naive subjects were as follows: the high absolute values of serum TXB_2_; the lack of influence of pharmacological interventions; and the possibility of detecting even small differences in absolute serum TXB_2_ values. The study was conducted in accordance with the Declaration of Helsinki and received ethics committee’s approvals in all participating centers.

Peripheral blood was withdrawn using a Vacutainer^®^ system into a Vacuette^®^ tube (Z Serum Clot Activator, Geiner Bio-One GmbH, Kremsmünster, Austria). Physicians and nurses were instructed to place the tubes within 3 min maximum after blood withdrawal into a 37 °C water bath located in the proximity of the outpatient Unit. After 1-h incubation, all blood samples were centrifuged at 1200 × *g* for 10 min, the serum supernatant was collected and stored at −20 °C until shipment. All centers recorded the anonymized subject ID, the timing of blood sampling, start and end of incubation, and storage at 20 °C in a data sheet. All samples were shipped frozen to a Core Lab, where centralized measurements were performed. Serum TXB_2_ was measured by enzyme immunoassay (EIA) as previously described^17,23^. This EIA method has a limit of detection calculated as 80% *B*/*B*_0_ of 3 ± 2 pg/ml, an inter-assay coefficient of variation of 6% (*n* = 75 determinations), and has been validated against gas chromatography/mass spectrometry^17^.

The reference range of serum TXB_2_ values was calculated as the mean ± 1 SD of 101 serum samples from healthy volunteers (43% females, median age 33 [30–49, interquartile range] years) from previously published studies^17,22,39^, which were measured in the same laboratory (Dept. of Pharmacology, Catholic University School of Medicine, Rome, Italy), using the same pre- and post-analytical procedures^14^. We considered the inter-assay coefficient of variation, calculated as SD/mean × 100 of the same sample measured in different assays. Thus, given a mean serum TXB_2_ value of 295 ± 121 ng/ml, and 6% inter-assay variability, we considered as lower limit of the normal range a concentration of 184 ng/ml. We considered a center as compliant with the procedure if it provided at least 4 out of 5 samples measuring ≥184 ng/ml. Centers who provided ≥2 samples out of range were interviewed about the procedure and were asked to repeat blood sampling and the pre-analytical procedure a second time.

**Fig. 2:**
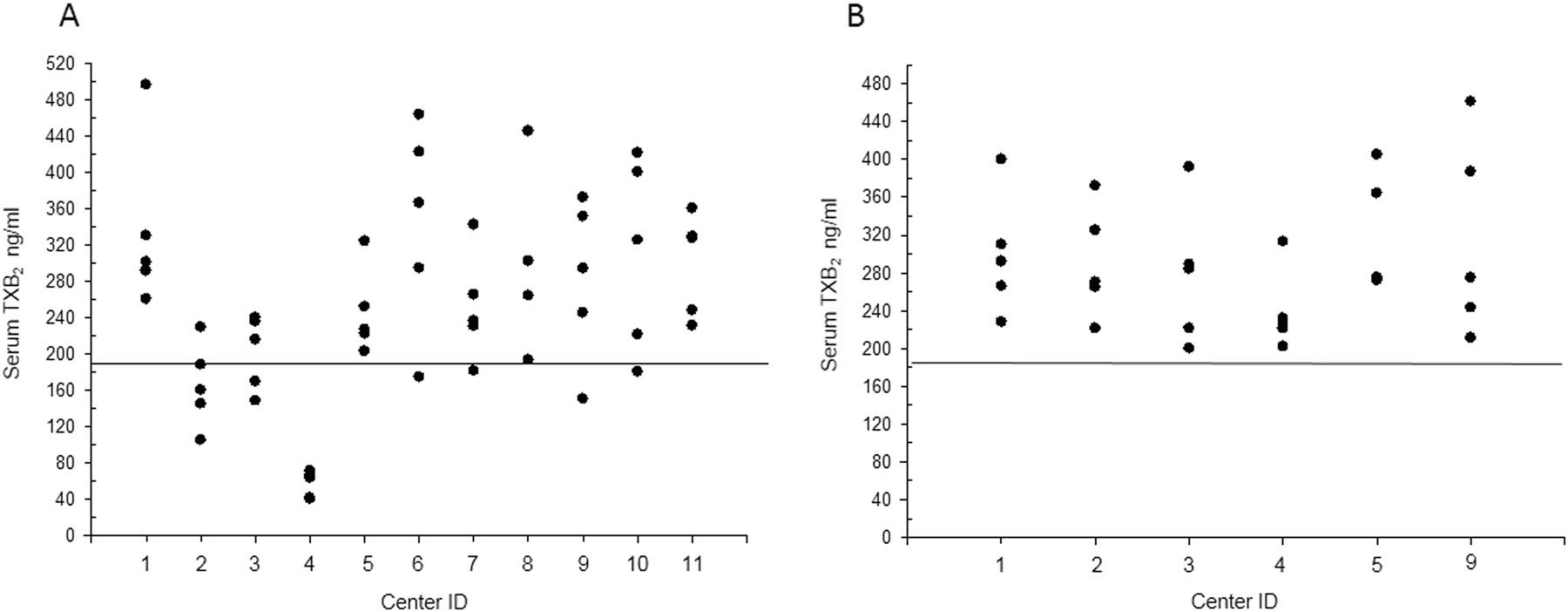
Individual serum TXB_2_ values across different ARES centers. **a** Individual serum TXB_2_ values measured in samples obtained from 55 healthy subjects in 11 centers. The lower limit of the normal range is indicated by the horizontal line. **b** The second serum TXB_2_ determination in 6 selected centers, with the 3 centers showing out-of-range values in the first set of determinations, and 3 other centers, which had appropriate values and were repeated for assessing data reproducibility. Center numbering is the same in **a** and **b**

Fifty-five healthy volunteers (60% females, median age 34 [29–48] years) were recruited in 11 centers. The logged time interval between blood sampling and 37 °C incubation was 1 [1–3.5] min (*n* = 55) and the time between the end of incubation of the samples and serum freezing was 31 [13–75] min (*n* = 55) without any statistically significant differences between centers. There was no correlation between each of these time intervals and the final serum TXB_2_ values (all *p* > 0.5). The serum TXB_2_ values of the first series of measurements are shown in Fig. 2a, and 3 out of 11 centers had ≥2 values ≤184 ng/ml. These centers were further queried regarding their procedures and instrumentation to assess the conditions of 37 °C incubation of the blood samples. One center used a dry heating instrument (cell incubator) rather than a water bath, to incubate whole blood (Fig. 2, center 4), one center had a water bath not reaching the correct temperature in spite of the displayed value (Fig. 2, center 2), one center used to wrap up the tubes with rubber before placing them in the water bath (Fig. 2, center 3). These conditions are likely to have caused an actual incubation temperature of the samples <37 °C or a delay in reaching the correct temperature in the sample. These three centers then modified their incubation conditions and repeated the procedure. As a control for internal reproducibiliy, three centers with appropriate serum TXB_2_ values repeated the procedure as well. Figure 2b shows the results of the second series of measurements in the six centers. All centers had values within the expected range (Fig. 2b).

## Conclusions

Despite considerable progress in understanding the pathophysiology of ET complications, substantial uncertainty remains concerning the optimal antiplatelet therapy, largely reflecting the following: (1) the lack of randomized clinical trials of antiplatelet prophylaxis in this setting; (2) the widely held assumption that a standard low-dose aspirin regimen is adequate for all ET patients, while in fact a od dosing regimen has been shown inadequate to achieve persistent inhibition of platelet TXA_2_ in the vast majority of ET patients^14,25^; (3) a substantial residual risk of major vascular events in spite of aspirin treatment^3,9^; and (4) a treatment recommendation of considering aspirin bid in low- to high-risk patients^1^, in the absence of a formal dose-finding study and efficacy trial.

The ARES study will be the first, multicenter, phase II randomized trial testing the hypothesis that the current standard antiplatelet regimen (low-dose aspirin od) is inadequate to ensure effective and persistent blockade of platelet COX-1 activity in ET patients, with the ultimate goal of optimizing antiplatelet therapy in intermediate- to high-risk ET patients who have a clear indication for long-term antiplatelet prophylaxis. ARES will provide essential information on the required dosing regimen to achieve this goal, as well as a preliminary assessment of its tolerability and safety that will inform the design of a properly sized phase III efficacy trial. The assessment of the reproducibility of the whole-blood TXB_2_ assay among centers, which we tested before starting patient enrollment, appears as an essential step to ensure the reliability of the main study results.

## References

[1] Tefferi A, Vannucchi AM, Barbui T (2018). Essential thrombocythemia treatment algorithm 2018. Blood Cancer J..

[2] Ryden L (2013). ESC guidelines on diabetes, pre-diabetes, and cardiovascular diseases developed in collaboration with the EASD-summary. Diab. Vasc. Dis. Res..

[3] De Stefano V (2008). Recurrent thrombosis in patients with polycythemia vera and essential thrombocythemia: incidence, risk factors, and effect of treatments. Haematologica.

[4] Vardiman JW (2009). The 2008 revision of the World Health Organization (WHO) classification of myeloid neoplasms and acute leukemia: rationale and important changes.. Blood.

[5] Deadmond MA, Smith-Gagen JA (2015). Changing incidence of myeloproliferative neoplasms: trends and subgroup risk profiles in the USA, 1973-2011. J. Cancer Res. Clin. Oncol..

[6] Moulard O (2014). Epidemiology of myelofibrosis, essential thrombocythemia, and polycythemia vera in the European Union. Eur. J. Haematol..

[7] Roaldsnes C, Holst R, Frederiksen H, Ghanima W (2017). Myeloproliferative neoplasms: trends in incidence, prevalence and survival in Norway. Eur. J. Haematol..

[8] Titmarsh GJ (2014). How common are myeloproliferative neoplasms? A systematic review and meta-analysis. Am. J. Hematol..

[9] Patrono C, Rocca B, De Stefano V (2013). Platelet activation and inhibition in polycythemia vera and essential thrombocythemia. Blood.

[10] Hultcrantz M (2015). Risk and cause of death in patients diagnosed with myeloproliferative neoplasms in Sweden between 1973 and 2005: a population-based study. J. Clin. Oncol..

[11] Rocca B (1995). Increased thromboxane biosynthesis in essential thrombocythemia. Thromb. Haemost..

[12] Dragani A (2009). The contribution of cyclooxygenase-1 and -2 to persistent thromboxane biosynthesis in aspirin-treated essential thrombocythemia: implications for antiplatelet therapy. Blood.

[13] Viallard JF (2002). Increased soluble and platelet-associated CD40 ligand in essential thrombocythemia and reactive thrombocytosis. Blood.

[14] Pascale S (2012). Aspirin-insensitive thromboxane biosynthesis in essential thrombocythemia is explained by accelerated renewal of the drug target. Blood.

[15] Patrono C (1986). J. Clin. Invest..

[16] Eikelboom JW (2002). Aspirin-resistant thromboxane biosynthesis and the risk of myocardial infarction, stroke, or cardiovascular death in patients at high risk for cardiovascular events. Circulation.

[17] Petrucci G (2016). Patient-independent variables affecting the assessment of aspirin responsiveness by serum thromboxane measurement. Thromb. Haemost..

[18] Alvarez-Larran A (2010). Observation versus antiplatelet therapy as primary prophylaxis for thrombosis in low-risk essential thrombocythemia. Blood.

[19] Alvarez-Larran A (2016). Antiplatelet therapy versus observation in low-risk essential thrombocythemia with a CALR mutation. Haematologica.

[20] Landolfi R (2004). Efficacy and safety of low-dose aspirin in polycythemia vera. N. Engl. J. Med..

[21] Patrono C, Garcia Rodriguez LA, Landolfi R, Baigent C (2005). Low-dose aspirin for the prevention of atherothrombosis. N. Engl. J. Med..

[22] Santilli F (2009). Platelet cyclooxygenase inhibition by low-dose aspirin is not reflected consistently by platelet function assays: implications for aspirin “resistance”. J. Am. Coll. Cardiol..

[23] Patrono C (1980). Low dose aspirin and inhibition of thromboxane B2 production in healthy subjects. Thromb. Res..

[24] Antithrombotic Trialists C (2009). Aspirin in the primary and secondary prevention of vascular disease: collaborative meta-analysis of individual participant data from randomised trials. Lancet.

[25] Dillinger JG (2012). Twice daily aspirin to improve biological aspirin efficacy in patients with essential thrombocythemia. Thromb. Res..

[26] Rocca B, Patrono C (2015). Platelet progenitors: the hidden drug target. Eur. Heart J..

[27] Cavalca V (2017). On-pump cardiac surgery enhances platelet renewal and impairs aspirin pharmacodynamics: effects of improved dosing regimens. Clin. Pharmacol. Ther..

[28] Rocca B (2012). The recovery of platelet cyclooxygenase activity explains interindividual variability in responsiveness to low-dose aspirin in patients with and without diabetes. J. Thromb. Haemost..

[29] Newby LK (2006). Long-term adherence to evidence-based secondary prevention therapies in coronary artery disease. Circulation.

[30] Diener HC (1996). European stroke prevention study. 2. Dipyridamole and acetylsalicylic acid in the secondary prevention of stroke. J. Neurol. Sci..

[31] Davi G, Patrono C (2007). Platelet activation and atherothrombosis. N. Engl. J. Med..

[32] FitzGerald GA, Brash AR, Oates JA, Pedersen AK (1983). Endogenous prostacyclin biosynthesis and platelet function during selective inhibition of thromboxane synthase in man. J. Clin. Invest..

[33] Cavalca V (2014). In vivo prostacyclin biosynthesis and effects of different aspirin regimens in patients with essential thrombocythaemia. Thromb. Haemost..

[34] Schulman S, Kearon C, Subcommittee on Control of Anticoagulation of the S, Standardization Committee of the International Society on T, Haemostasis. (2005). Definition of major bleeding in clinical investigations of antihemostatic medicinal products in non-surgical patients. J. Thromb. Haemost..

[35] Kaatz S, Ahmad D, Spyropoulos AC, Schulman S, Subcommittee on Control of A. (2015). Definition of clinically relevant non-major bleeding in studies of anticoagulants in atrial fibrillation and venous thromboembolic disease in non-surgical patients: communication from the SSC of the ISTH. J. Thromb. Haemost..

[36] Rabeneck L (2002). Reliability, validity, and responsiveness of severity of dyspepsia assessment (SODA) in a randomized clinical trial of a COX-2-specific inhibitor and traditional NSAID therapy. Am. J. Gastroenterol..

[37] Scherber R (2011). The Myeloproliferative Neoplasm Symptom Assessment Form (MPN-SAF): international prospective validation and reliability trial in 402 patients. Blood.

[38] Arber DA (2016). The 2016 revision to the World Health Organization classification of myeloid neoplasms and acute leukemia. Blood.

[39] Zaccardi F (2016). In vivo platelet activation and aspirin responsiveness in type 1. Diabetes.

[40] Barbui T (2011). Survival and disease progression in essential thrombocythemia are significantly influenced by accurate morphologic diagnosis: an international study. J. Clin. Oncol..

[41] Finazzi G (2012). Incidence and risk factors for bleeding in 1104 patients with essential thrombocythemia or prefibrotic myelofibrosis diagnosed according to the 2008 WHO criteria. Leukemia..

[42] Patrono C (1985). Clinical pharmacology of platelet cyclooxygenase inhibition. Circulation.

[43] Holinstat M (2011). Protease-activated receptor signaling in platelets activates cytosolic phospholipase A2alpha differently for cyclooxygenase-1 and 12-lipoxygenase catalysis. Arterioscler. Thromb. Vasc. Biol..

[44] Patrignani P, Filabozzi P, Patrono C (1982). Selective cumulative inhibition of platelet thromboxane production by low-dose aspirin in healthy subjects. J. Clin. Invest..

[45] Frelinger AL (2009). Association of cyclooxygenase-1-dependent and -independent platelet function assays with adverse clinical outcomes in aspirin-treated patients presenting for cardiac catheterization. Circulation.

[46] Reny JL (2012). Antiplatelet drug response status does not predict recurrent ischemic events in stable cardiovascular patients: results of the Antiplatelet Drug Resistances and Ischemic Events study. Circulation.

[47] Brun C (2016). Platelets.

[48] van Diemen JJK (2018). Influence of pre-analytical time and temperature conditions on serum thromboxane B2 levels. Thromb. Res..

